# SARS/MERS/SARS-CoV-2 Outbreaks and Burnout Syndrome among Healthcare Workers. An Umbrella Systematic Review

**DOI:** 10.3390/ijerph18084361

**Published:** 2021-04-20

**Authors:** Nicola Magnavita, Francesco Chirico, Sergio Garbarino, Nicola Luigi Bragazzi, Emiliano Santacroce, Salvatore Zaffina

**Affiliations:** 1 Post-Graduate School of Occupational Medicine, Università Cattolica del Sacro Cuore, 00168 Rome, Italy; nicola.magnavita@unicatt.it (N.M.); sgarbarino.neuro@gmail.com (S.G.); emiliano.santacroce@aslroma5.it (E.S.); 2 Department of Woman/Child and Public Health, Fondazione Policlinico “A. Gemelli” IRCCS, 00168 Rome, Italy; 3 Health Service Department, State Police, Ministry of Interior, 20125 Milan, Italy; 4 Department of Neuroscience, Rehabilitation, Ophthalmology, Genetics, Mother and Child Health (DINOGMI), University of Genoa, 16132 Genoa, Italy; 5 Laboratory for Industrial and Applied Mathematics (LIAM), Department of Mathematics and Statistics, York University, 4700 Keele Street, Toronto, ON M3J 1P3, Canada; bragazzi@yorku.ca; 6 Workplace Prevention and Safety Service, Local Sanitary Unit Roma 5, 00012 Guidonia Montecelio, Italy; 7 Occupational Health Unit, Medical Direction, Bambino Gesù Children’s Hospital IRCCS, 00165 Rome, Italy; salvatore.zaffina@opbg.net

**Keywords:** burnout syndrome, mental health, coronavirus, COVID-19, healthcare, occupational stress, prevention

## Abstract

The coronavirus-19 (COVID-19) pandemic is putting a severe strain on all healthcare systems. Several occupational risk factors are challenging healthcare workers (HCWs) who are at high risk of mental health outcomes, including Burnout Syndrome (BOS). BOS is a psychological syndrome characterized by emotional exhaustion, depersonalization, and low personal accomplishment. An umbrella review of systematic reviews and meta-analyses concerning BOS and coronavirus (SARS/MERS/SARS-CoV-2) outbreaks was carried out on PubMed Central/Medline, Cochrane Library, PROSPERO, and Epistemonikos databases. Data relating to COVID-19 is insufficient, but in previous SARS and MERS outbreaks about one-third of HCWs manifested BOS. This prevalence rate is similar to the figure recorded in some categories of HCWs exposed to chronic occupational stress and poor work organization during non-epidemic periods. Inadequate organization and worsening working conditions during an epidemic appear to be the most likely causes of BOS. Preventive care and workplace health promotion programs could be useful for protecting healthcare workers during pandemics, as well as during regular health activities.

## 1. Introduction

The ongoing COVID-19 pandemic resulting from the new SARS-CoV-2 virus has exposed healthcare workers (HCWs) to overwhelming pressure. In an efficient healthcare system, priority should be given to protecting healthcare workers [1], but on 26 February 2021, the COVID-19 integrated surveillance data in Italy, carried out by the National Health Institute, indicated that there have been 123,025 cases among HCWs since the beginning of the COVID-19 pandemic, accounting for 4.2% of the total number of cases registered in the country [2]. Furthermore, 81 deaths were reported among Italian nurses [3] and 326 among doctors [4]. A systematic literature search found that, as of 8 May 2020, more than 152,000 infections and 1413 deaths in HCWs had been reported, worldwide. Over 70% of the infections were in women, with nurses accounting for almost 40% of the total, whereas mortality occurred mainly in men (70.8%), with doctors accounting for 51.4% of deaths [5]. Lack of personal protective equipment (PPE), unprotected exposure to infected patients, work overload, and pre-existing medical conditions were identified as major risk factors in a situation that presented an unprecedented occupational risk of morbidity and mortality [6].

Moreover, the pandemic has put particular psychological pressure on understaffed and poorly supplied services. A significantly increased risk of anxiety, depression, and sleep disturbances was found in HCWs who had experienced unprotected exposure to patients with COVID-19, and especially in those who had contracted the infection [7]. Frontline HCWs experienced the negative consequences of inadequate healthcare management and were exposed to overwork, high emotional demands, excessive effort, and reduced rewards. Less time for physical activities, meditation, and relaxation increased work-related stress [8]. Compassion fatigue, uncertainty about therapies and safety procedures [8], ethical dilemmas, and deprivation of family during quarantine [9] are among the stressors associated with the pandemic. Overworking and emotional distress can lead to an increase in errors and a reduction in the quality of care [10,11]. This, in turn, may provoke litigation [12] and consequently increase occupational stress.

The first wave of the COVID-19 pandemic was associated with a high incidence of anxiety, sleep problems, depression, and post-traumatic stress disorder in HCWs [13,14,15]. With successive waves of contagion that are continually challenging health systems, more insidious mental health problems, such as burnout, have emerged [16].

Burnout syndrome (BOS) is a psychological syndrome characterized by factors such as emotional exhaustion (EE), mental fatigue, depersonalization (DP) or cynicism in the form of negative feelings and perceptions about the people one works with, and low personal accomplishment (LPA) [17,18,19]. In the 11th version of the International Classification of Diseases (ICD-11), the World Health Organization (WHO) included BOS as “a syndrome resulting from chronic workplace stress that has not been successfully managed” [20].

Even prior to the current pandemic, the high prevalence of BOS among HCWs was widely reported as a serious health problem for the world economy, due to its adverse effects on patients and organizational outcomes [21]. BOS has also been associated with anxiety, depression, lower satisfaction, and care quality, as well as Post Traumatic Stress Disorder and an increased suicide rate among HCWs [22,23]. It was found that the COVID-19 pandemic presents a sort of perfect storm regarding the intersection of chronic workplace stress resulting in high rates of HCWs burnout and acute traumatic stress imposed by the pandemic [24]. It is intuitive that the pandemic increases HCWS’s workload, which, in turn, increases BOS; however, exposure to COVID-19 infection does not necessarily correlate with an increased risk of BOS [25]. A large number of factors interact in various ways in the workplace; among other things, all pre-existing inequalities can be accentuated during the pandemic, for example, gender inequities can be enhanced in times of pandemic [26]. In the fight against COVID-19, BOS has been described as a major threat to the stability of the frontline workforce [27,28,29,30]. The confrontation with a new, highly diffusive disease exposes workers to significant stress that can affect mental health in different ways; acute stress will mainly cause anxiety, mood changes, sleep disorders, fear, and post-traumatic stress disorder, while chronic stress may cause BOS, which is a condition strongly linked to impaired work ability [31,32]. The health worker suffering from BOS is unmotivated, exhausted, and cynical, and therefore, can be very dangerous for patients. Indeed, burned-out healthcare workers are not able to provide high-quality healthcare services. Unfortunately, until now, no significant steps have been taken by the occupational stakeholders to minimize the COVID-19 risk factors for BOS in HCWs [33].

All these considerations indicate the need to assess the risk of BOS in HCWs on the basis not only of the empirical evidence that is currently accumulating, but also on the well-established evidence derived from studies conducted during previous outbreaks of coronavirus, namely, SARS and MERS (Middle East Respiratory Syndrome) epidemics. In this way, the information needed for supporting effective preventive measures will be immediately available.

The main aim of this umbrella review was, therefore, to summarize and combine relevant data from existing systematic reviews and meta-analyses in order to evaluate the association of coronaviruses (SARS, MERS, SARS-CoV-2) with BOS in HCWs. A further aim was to provide clinical decision makers with the evidence they need for targeted interventions to protect the mental health of HCWs.

## 2. Materials and Methods

### 2.1. Information Sources and Search

From 31 October 2020 to 31 March 2021, using the “systematic review” filter, a search was conducted on PubMed Central and Medline with combinations of the following keywords and synonyms in conjunction with the controlled vocabulary of the database: “healthcare”, “physician”, “burnout”, “COVID-19”, “SARS”, “MERS”, and “SARS-CoV-2”. Specific repositories of systematic reviews such as the JBI Database of Systematic Reviews (The Joanna Briggs Institute, Adelaide, Austrilia), the Cochrane Library (Cochrane, London, UK), PEDro (PEDro Partnership, Institute for Musculoskeletal Health, The University of Sydney, Sydney, Australia), OT Seeker (Department of Occupational Therapy, The University of Queensland, Brisbane, Australia), PROSPERO (National Institute for Health Research, University of York, York, UK), and federated search engines such as TRIP (Trip Database Ltd. Company, London, UK), DARE (National Institute for Health Research, University of York, York, UK), and Epistemonikos (Epistemonikos Foundation, Providencia, Santiago, Chile) were also checked. Due to the rapid production of publications during the current pandemic, pre-print papers were also searched on MedXriv (BMJ, Yale, UK).

### 2.2. Eligibility Criteria

Criteria of inclusion were the following: 1. Systematic review studies which examined the occurrence of burnout in HCWs during or after SARS, MERS, and COVID-19 outbreaks. 2. Available in English language. 3. Published before 31 March 2021.

Criteria of exclusion were: 1. Studies written in non-English languages. 2. Non-systematic reviews. 3. Studies without quantitative data on burnout.

Only systematic reviews, that were published in English, containing a quantitative analysis of the results, and investigating the prevalence of BOS in HCWs during major coronavirus outbreaks and epidemics, were included. Editorials, original research, commentaries, narrative reviews, and dissertations were excluded. To reduce the risk of bias, two independent reviewers (FC, NB) screened the total list of identified records to determine eligibility and a third reviewer (NM) was available to resolve eventual disagreements. Screening was initially via title and then abstract. FC also searched the full reference lists of selected studies and identified pre-print papers on MedXriv.

### 2.3. Data Collection

Two researchers (FC, SG) independently extracted data from all the included studies. Discrepancies were resolved through consensus, under the supervision of a senior researcher (NM). A summary of selected variables included first author and year of publication, type of strategy used, method adopted to assess the quality of the studies, and the number and type of studies included in each review.

## 3. Results

Of the 270 records, 16 systematic reviews were read and seven were included in this review (see Table 1). Two studies were excluded because they were published in Spanish and two systematic reviews of qualitative studies were excluded because they focused on negative emotions and emotional control but did not concern emotional exhaustion or burnout. One study conducted during the COVID-19 pandemic in HCWs was excluded because BOS was not included among the psychological outcomes. Three further systematic reviews were excluded because they had different systematic reviews design (e.g., integrative, scoping, and mixed-method systematic review). One study was excluded because it concerned the measures against BOS in times of COVID-19.

The systematic review by Chew et al. [34] included 23 papers (15 quantitative and eight qualitative reviews). Overall, 17 studies examined the SARS epidemic, five focused on Ebola epidemics, and one covered the MERS epidemic; no study investigated the SARS-CoV-2 epidemic. Chew identified several psychological responses, including burnout, that were related to, and persisted after the epidemics. Other psychological consequences were anxiety, fears, stigmatization, depression, post-traumatic stress, anger/frustration, and grief. HCWs exposed to infected patients reported significantly higher levels of burnout than their non-exposed colleagues [35]. Burnout and specifically emotional exhaustion were associated with having more contact with infected patients, lower levels of stamina, and less confidence in infection control initiatives [36]. Individual coping strategies, such as seeking social support, positive thinking, problem solving, and avoidance, added to institutional intervention that included guidance and training on infection control and the use of equipment; leadership support at the workplace and psychosocial support in terms of psychiatric help, monitoring, or clinical supervision; or a staff buddy system provided by the hospital administration, were found to boost lower rates of emotional exhaustion [36].

The systematic review with meta-analysis conducted by Serrano-Ripoll [37] examined three relevant areas: the prevalence of mental health problems (including BOS), the factors associated with an increased likelihood of developing those problems, and the effects of interventions for improving the mental health of HCWs. This review included 113 studies that examined the impact of epidemics on mental health and four studies that investigated interventions to reduce that impact. The authors calculated a pooled prevalence of BOS, based on a study conducted during the H1N1A virus epidemic in Mexico and two studies on SARS—one performed during, and one soon after the epidemic. The estimated pooled prevalence was 28% (95% CI 26 to 31%, 3 studies, 1,168 participants). No data was available to ascertain potential gender differences in the prevalence of BOS. According to the studies retrieved in this review, symptoms mainly affected nurses [38] and residents [39]. Lack of support from family and friends was also found to be associated with BOS [40]. Furthermore, lack of specialized training, working in a high-risk environment, and being in direct contact with infected patients were also risk factors for BOS.

The systematic review by Salazar de Pablo et al. [41] addressed the effects of SARS/MERS/CoV-2 on both the physical and mental health of HCWs and made a meta-analysis of the outcomes. The pooled prevalence of BOS in HCWs during epidemics was estimated at 34.4% (95% CI = 19.3–53.5%). Of the 115 studies on mental outcomes in HCWs during epidemics, only three studies focused on BOS; of these, two studies (1305 cases) related to SARS, one (32 cases) to Covid-19. The studies included in this meta-analysis were different from those selected by Serrano et al. [37].

The aforementioned systematic analyses include very few studies on BOS during the current COVID-19 pandemic. In the two meta-analyses on the prevalence of burnout, the only study on workers struggling with COVID-19 was based on a mere 32 subjects. Therefore, not enough data is available to evaluate the association between BOS and COVID-19. However, the three systematic studies concur in reporting that a significant proportion of HCWs exhibit BOS symptoms during viral outbreaks. The pooled prevalence estimated in the aforementioned meta-analyses ranged from 28% (95% CI = 25–31%) [37] to 34.4% (95% CI = 19.3–53.5%) [41].

The obvious relevance of the topic has led various research groups to produce summary studies on the copious research concerning HCWs struggling with COVID-19, or to re-analyze data collected in previous SARS and MERS outbreaks. The systematic review by Sanghera et al. [42], which was performed between 31st December 2019 and 17th June 2020, reported five studies on BOS in frontline HCWs; prevalence rates of BOS ranged between 3.1% and 43%.

The review by Danet Danet [43], online in March 2021, lists twelve cross-sectional studies conducted in Western countries on the mental health of HCWs facing COVID-19. Two of these studies were conducted in Italy and reported the presence of BOS, with higher levels of EE and DP among women, nurses, and younger workers who had a greater workload.

A systematic review and meta-analysis, published in March 2021 [44], identified 16 studies on nurses working with COVID-19 patients. The estimated prevalence of EE was 34.1% (95% CI = 22.5–46.6%), of DP 12.6% (95% CI = 6.9–19.7%), and of LPA 15.2% (95% CI = 1.4–39.8%). A considerable heterogeneity (*I*^2^ ranging from 98.9% and 99.8%) was observed. Studies included in this review were all cross-sectional; many of them did not account for confounding factors and did not apply multivariable methods to eliminate them. Furthermore, many of them did not define in-detail criteria followed for inclusion in the sample, settings, and characteristics of study subjects.

Busch et al. [45], in a study published online in February 2021, estimated a pooled prevalence of BOS of 31.81% (95% CI = 13.32–53.89) in frontline HCWs during SARS, MERS, and COVID-19 outbreaks. In this study, however, a high heterogeneity (*I*^2^ index = 99.77%) was found. When a considerable heterogeneity is found, according to the Cochrane Handbook [46], using meta-analysis may produce misleading results. The best choice, in the presence of identifiable reasons for heterogeneity (e.g., clinical differences between diseases, geographic, socio-economic, and ethnic differences, etc.), could be the use of subgroup analysis or meta-regression.

## 4. Discussion

The main observation that emerges from this umbrella review is that in previous outbreaks of SARS and MERS, about one-third of HCWs experienced BOS, while data on the relationship between COVID-19 and BOS in HCWs is only forming now. The first available studies on HCWs addressing COVID-19 seem to confirm prevalence rates of BOS similar to those found in previous epidemics. However, it is probable that the pandemic, due to the fact of involving peoples of very different economic, political, and health status, and the longer duration of the epidemic, could have different and potentially more serious effects than those observed in the previous coronavirus epidemics. This is not surprising since burnout is a pathology that is the result of chronic exposure to stress and in the first wave of the COVID-19 epidemic there was not enough time to detect this disorder. Nevertheless, studies conducted in previous coronavirus outbreaks have reported the presence of BOS in HCWs who had to cope with those epidemics.

HCWs are exposed to numerous stressors during a pandemic. Past experience has shown that protracted epidemics and the unfavorable conditions, in which HCWs operate for a long time, favor the emergence of chronic effects of stress, such as depression and burnout. The continuing duration of the COVID-19 pandemic raises fears that all the events witnessed in the past may occur again in this emergency. For this reason, a careful analysis of published studies is of interest since it can help us to use past experiences to better understand what may happen today.

A number of on-going studies during the COVID-19 pandemic have recently been published and it is commonly believed that BOS can occur in HCWs during a pandemic. Studies have highlighted the presence of BOS in workers struggling with COVID-19 patients. HCWs include frontline nurses in emergency departments [47] and ICUs [48], Libyan [49] or Indian [50] physicians, Spanish nurses [51], Japanese radiological technologists and pharmacists [52], and others. A state of persistent burnout, that influenced sleep quality, was observed in a longitudinal study on a small group of HCWs [53]. Higher levels of stress [54] and long shifts [55] have been consistently associated with increased levels of BOS in HCWs.

However, some studies have found a reduction in BOS during the COVID-19 outbreak: for example, in US neurosurgeons [56], and in Chinese frontline nurses, compared with ordinary ward workers [57]. A relatively low level of burnout was also observed in healthcare workers in French geriatric facilities providing acute care [58]. There was no association between burnout and exposure to the consequences of COVID-19 among French paediatric residents [59]. In studies conducted so far, there are conflicting findings on the epidemiology of BOS among HCWs working in COVID-19 wards [16]. A pandemic generates very different working conditions in the various countries according to the different phases of the epidemic and the different economic, social, organizational, and health conditions. A thorough understanding of what has happened, or is happening, will evidently require further study. The promptly published review studies had to deal with studies of high heterogeneity and, often, of not very good quality. The explosion of the pandemic, in fact, has caused enormous pressure to publish and this has often put the systems of revision of scientific publications [60] in crisis. The so-called “infodemics” can be countered with high-quality reviews, inspired by rigorous selection criteria [61].

In view of such a complex picture, the question arises as to whether all BOS cases observed are attributable to epidemics, or whether some existed prior to epidemics and were due to common health activity problems.

In general, an association between two phenomena is not sufficient to demonstrate an etiological link. The classic Henle-Koch postulates developed for viral diseases, and for causative agents in chronic diseases, have been discussed and reviewed in terms of their full validity [62]. The main limitation of postulates is that they do not consider the possibility of a multiple etiology and do not take into account other factors in addition to the one studied. Associating coronaviruses with BOS might only be a simplistic approach that does not consider the possibility of other environmental factors that may lead to BOS. In the course of their normal healthcare activities, HCWs are often exposed to numerous occupational stressors that continue to exert their effect during pandemics. Studies conducted during an epidemic often adopt very elementary survey models that are unable to consider all the factors involved.

Most of the studies selected in the aforementioned reviews were cross-sectional or, at most, retrospective. Consequently, the effects of an epidemic could not be disentangled from those of other factors that cause BOS. Only longitudinal studies demonstrating the increase in BOS or the psychological phenomena that comprise it (EE, DP, and LPA) during an epidemic/pandemic are able to provide this evidence. However, because of the sudden onset of epidemics, it has never been possible to plan longitudinal investigations with a pre and post design capable of ascertaining how BOS evolves.

As an alternative, researchers could use information from repeated cross-sectional studies that compare the prevalence of BOS in a given population before, during, and after the end of an epidemic/pandemic. Unfortunately, no such studies were carried out for previous coronavirus epidemics. To fill this gap, a national, repeated cross-sectional study has been planned in Thailand to describe the mental health status and psychosocial problems arising among HCWs during the current COVID-19 pandemic [63].Another smaller study concerning a COVID-19 hub hospital in Italy has already published the baseline data collected during the 1st wave of the pandemic [8].

In order to interpret the high frequency of BOS observed in HCWs during an epidemic, it may be useful to compare this finding with the prevalence reported in studies on HCWs during periods of normal working conditions. However, some uncertainty exists regarding the prevalence of BOS among HCWs and associations between BOS and the gender, age, specialty, and geographical location of HCWs [64]. Elevated BOS levels have been reported in some subgroups of HCWs regardless of epidemic outbreaks. In a systematic review with meta-analysis performed in 2019, Low et al. [64]. estimated an aggregate prevalence of BOS of 51.0% (95% CI = 45.0–57.0%) in residents. Molina-Praena et al., in a 2018 meta-analytic study on medical nurses, estimated a pooled prevalence of 31% for EE, of 24% for DP, and of 38% for LPA, in this category of workers. [65]. In a 2017 meta-analysis on emergency nurses [66], the estimated prevalence of EE was 31% (95% CI = 20–44), 36% (95% CI = 23–51) for DP, and 29% (95% CI = 15–44) for LPA. In palliative care nurses, the estimated meta-analytic prevalence was 24% (95% CI = 16–34%) for EE, 30% (95% CI = 18–44%) for DP, and 28% for LPA [67]. A meta-analytic study on oncologists estimated the pooled prevalence rates for MBI subscales of EE at 32%, DP at 24%, and LPA at 37% [68]. A review of the literature, therefore, allows us to state that the prevalence of BOS observed in HCWs struggling with epidemics is similar to that found in other categories of HCWs, such as residents, oncologists, and nurses in palliative care.

In conclusion, current evidence has failed to find a strong association between BOS and an epidemics/pandemic in HCWs. Nevertheless, the presence of numerous stress factors during an outbreak of infectious disease makes the association between a pandemic and BOS highly plausible. A systematic review of prospective studies demonstrated that high psychosocial demands, high workload, low job control, low reward, and job insecurity increased the risk of developing BOS in HCWs [69]. Since all these stress factors are associated with an epidemic/pandemic, we believe that even when definitive evidence is lacking, a precautionary principle calls for every effort to be made to prevent BOS in HCWs.

Burnout in healthcare is an increasingly important pervasive phenomenon. An analysis of high-quality prospective studies has demonstrated that BOS is a significant predictor of many physical and psychological outcomes, such as metabolic syndrome and cardiovascular disorders, musculoskeletal disorders, fatigue, headaches, gastrointestinal and respiratory problems, severe injuries and early mortality, insomnia, depression, job dissatisfaction, absenteeism, and presenteeism [22]. Neglecting the risk of BOS can lead to a damaging series of consequences with progressively negative effects on the health of workers and the quality of care. Since it is vital to tackle the mental health problems of HCWs during this pandemic in order to safeguard the functioning capacity of healthcare systems [70], occupational health promotion programs have been proposed to support these workers. Governments, policymakers, and relevant stakeholders should not only monitor and follow outcomes, but also conduct scientifically sound interventional research in order to mitigate the mental health impact on HCWs. In conjunction with institutional measures, numerous interventions for enhancing individual coping responses have been put forward to prevent the onset of BOS [71,72]. Workplace health programs could include individual interventions, based on psychoeducation, adequate sleep and rest in between workplace duties and shifts, the maintenance of social relationships, and improvements in problem-solving skills. In order to prevent BOS and other mental outcomes, healthcare policymakers could also consider the importance of providing organizational support, such as clear communication of changes, access to resources for psychological support, the empowerment of self-help groups, and the early identification of “at-risk” individuals. Once again, both a quantitative and qualitative analysis of evidence is lacking. The COVID-19 pandemic offers an excellent opportunity for developing mental health promotion activities that would lead to a correct and neutral ratification of findings. New studies carried out with a pre and post design during an epidemic/pandemic could provide useful information for selecting interventions that are most beneficial for the resilience and mental health of frontline healthcare workers [73].

A systematic analysis of the qualitative studies conducted on nurses during the COVID-19 pandemic indicates that the physical and emotional impact were derived from two categories, specifically; concerns for personal and family safety; and fear, vulnerability, and psychological issues in the face of crisis. Supportive interventions were mainly based on team’s sense of duty, work engagement and personal dedication, and professional collegiality [74]. Responsiveness of systematized, organizational reaction is of the utmost importance to control the emergence of BOS in HCWs.

Several studies have suggested various methods for preventing or reducing BOS. These methods can be divided into two categories: individual methods and organizational (system-based) approaches. At individual level, simple measures such as maintaining regular exercise, a balanced diet, and having a good rest, combined with a correct emotional balance, job satisfaction, family support, and happiness, are associated with a low frequency of BOS [59]. However, individual interventions are unlikely to be successful, unless accompanied by structural interventions. Health managers’ and policymakers’ awareness of BOS in HCWs is, therefore, important in stimulating and implementing preventive interventions. Improving work schedules, providing counseling support meetings that promote self-management, and mindfulness-based stress control activities are among the suggested techniques to prevent or reduce BOS in HCWs during the COVID-19 pandemic [59].

The systematic review of interventions aimed at supporting the resilience or mental health of frontline HCWs during SARS, MERS, or COVID-19 disease outbreaks [73] demonstrated that the lack of awareness of the need for psychological support and a lack of equipment, staff time, or skills needed for an intervention are the main factors hindering the prevention of BOS. Effective communication, safe and supportive environments for frontline workers, and careful attention for local needs, on the other hand, can promote the improvement of mental health and the prevention of BOS [73].

## 5. Conclusions

Studies conducted during the SARS and MERS epidemics find confirmation in the first observations conducted during the COVID-19 pandemic: a significant share of HCWs can develop BOS. Our study has the merit of underlining that it is simplistic to attribute the finding of EE, DP, and LPA to the coronavirus outbreak without delving into the numerous factors that can affect the phenomenon. Prospective or repeated cross-sectional studies will help to disentangle the etiological factors of the syndrome. In the meantime, however, preventive action must be taken.

BOS is the final stage of a prolonged state of work-related stress. HCWs who suffer from BOS during an epidemic/pandemic probably experienced low levels of satisfaction and wellbeing and high levels of occupational stress long before the epidemic/pandemic began. Mental health of HCWs should be improved, before the epidemic occurs, by promptly improving the working conditions of those in the frontline. As SARS/MERS/COVID-19 have a substantial impact on the mental health of HCWs, these interventions should become a priority for public health strategies.

This umbrella review has some limitations linked to the nature and quality of systematic reviews on BOS during epidemics. Since the aforementioned reviews did not contain longitudinal studies, it was not possible to analyze a variation before/after the epidemic. However, this problem was common to many review studies of mental health problems associated with COVID-19. In fact, most of the studies retrieved in these reviews were cross-sectional and often lacked a control group. Mental health symptoms in HCWs were generally compared to “normal values”, administrative staff, or external samples. The paradoxical result is that, despite the numerous studies produced, there is still little evidence of an increase in mental health problems in HCWs during the COVID-19 pandemic [75]. New studies to be published shortly will probably reach more statistically and epidemiologically robust conclusions.

Preventing BOS in HCWs is always a pressing need during a pandemic, when the quality of health services is critical. The experience gathered in previous coronavirus epidemics, reinforced by the first data that emerge from the observation of workers struggling with Covid-19, leads us to believe that the implementation of prevention policies and interventions is an indelible need. We hope that this study will serve to stimulate the acquisition of this awareness by managers and stakeholders.

## Figures and Tables

**Table 1:** Systematic reviews included in the review (*n* = 7).

Author	Type of Study	Search Strategy	Risk of Bias (Quality) Assessment	Studies Included	Main Findings
Salazar de Pablo et al. (2020)	Systematic review and meta-analysis	Web of Science database (Clarivate Analytics) was searched, incorporating the Web of Science Core Collection, the BIOSIS Citation Index, the KCI-Korean Journal Database, MEDLINE^®^, the Russian Science Citation Index, and the SciELO Citation Index, from inception until 15th April 2020.	A modified version of the Mixed Methods Appraisal Tool (MMAT)	115 studies, of which, 65 focused on SARS, 26 on MERS, and 24 on COVID-19. 3 studies on BOS	34.4% HCW exposed to SARS/MERS/COVID-19 reported burnout (95% CI = 19.3–53.5%, k = 3, *n* = 1337)
Chew et al. (2020)	Systematic review	PubMed, MEDLINE (Ovid), and Web of Science, combining key terms regarding recent infectious disease outbreaks and psychological and coping responses. Papers published from database inception to 20 April 2020, were considered for inclusion	The McMaster University critical appraisal tool was used to appraise quantitative studies. The guidelines by Higginbotham and colleagues were used to appraise qualitative studies.	23 studies, of which, 2 studies on BOS	HCW exposed to infected patients reported significantly higher levels of BOS than their colleagues who were not. BOS, and specifically EE, was predicted by having more contact with infected patients, lower levels of vigor, and less trust in infection control initiatives
Serrano-Ripoll (2020)	Rapid systematic review and meta-analysis	MEDLINE, Embase, and PsycINFO (inception to August 2020).	Evidence Partners (McMaster University) (Partners, 2020) tools for observational studies and ROBINS I (Sterne et al., 2016) for uncontrolled trials. GRADE to ascertain the certainty of evidence.	117 studies (3 studies on BOS, of which, 2 during the viral epidemic, and 1 after the viral epidemic)	The pooled prevalence for BOS was 28% (26 to 31%)
Sanghera et al. (2020)	Systematic review	MEDLINE and Embase. Papers published from 31 December 2019 to17 June 2020.	Method of assessment not indicated.	44 cross-sectional studies (of which, 5 on BOS)	BOS prevalence ranged between 3.1% and 43.0%. Nursing profession, being residents and younger age, were risk factors for BOS.
Danet Danet (2021)	Systematic review	PubMed, Scopus, and Web of Science from the beginning of the COVID-19 pandemic to 6 August 2020	Good practices standard criteria for questionnaire-based, cross-sectional, quantitative studies.	12 cross-sectional studies, of which, 2 on BOS	BOS associated with greater workload, younger age, female gender, nursing personnel, and related to a worse self-perceived state of health.
Galanis et al. (2021)	System-atic re-view and meta-analysis	PubMed, Scopus, ProQuest, Cochrane COVID-19 Registry, CINAHL, pre-print services (medRxiv, PsyArXiv) from 1 January to 15 November 2020	The 8-item/11-item Joanna Briggs Institute Critical Appraisal Checklist for Cross-sectional/cohort studies	16 cross-sectional studies on BOS	Pooled prevalence of EE 34.1% (95% CI 22.5–46.6%), DP 12.6% (95% CI 6.9–19.7%), LPA 15.2% (95% CI 1.4–39.8%) in nurses (6 studies). Association of BOS with sociodemographic factors (gender, age, educational level, and degree), social, and occupational factors
Busch et al. (2021)	Systematic review and meta-analysis	PubMed, Web of Science, MEDLINE, PsycINFO (inception to 19 March 2020)	The 8-item/11-item Joanna Briggs Institute Critical Appraisal Checklist for Cross-sectional/cohort studies	86 studies on psychological symptoms in frontline HCWs during SARS, H1N1, Ebola, MERS, COVID-19.	Pooled prevalence of BOS 31.81 (95% CI 13.32–53.89)

## Data Availability

The data presented in this study are available on request from the corresponding author.
